# Portable Respiration Monitoring System with an Embroidered Capacitive Facemask Sensor

**DOI:** 10.3390/bios12050339

**Published:** 2022-05-15

**Authors:** Mitar Simić, Adrian K. Stavrakis, Ankita Sinha, Velibor Premčevski, Branko Markoski, Goran M. Stojanović

**Affiliations:** 1 Faculty of Technical Sciences, University of Novi Sad, 21000 Novi Sad, Serbia; mitar.simic@uns.ac.rs (M.S.); sadrian@uns.ac.rs (A.K.S.); ankita.sinha@uns.ac.rs (A.S.); sgoran@uns.ac.rs (G.M.S.); 2 Technical Faculty Mihajlo Pupin, University of Novi Sad, 21000 Zrenjanin, Serbia; velibor.premcevski@tfzr.rs

**Keywords:** capacitive sensor, machine embroidery, e-textile, facemask, respiration monitoring, sensor-microcontroller interface

## Abstract

Respiration monitoring is a very important indicator of health status. It can be used as a marker in the recognition of a variety of diseases, such as sleep apnea, asthma or cardiac arrest. The purpose of the present study is to overcome limitations of the current state of the art in the field of respiration monitoring systems. Our goal was the development of a lightweight handheld device with portable operation and low power consumption. The proposed approach includes a textile capacitive sensor with interdigitated electrodes embroidered into the facemask, integrated with readout electronics. Readout electronics is based on the direct interface of the capacitive sensor and a microcontroller through just one analog and one digital pin. The microcontroller board and sensor are powered by a smartphone or PC through a USB cable. The developed mobile application for the Android™ operating system offers reliable data acquisition and acts as a bridge for data transfer to the remote server. The embroidered sensor was initially tested in a humidity-controlled chamber connected to a commercial impedance analyzer. Finally, in situ testing with 10 volunteering subjects confirmed stable operation with reliable respiration monitoring.

## 1. Introduction

Emerging wearable electronics technologies have received huge interest in scientific and professional communities in recent decades [1,2]. Advantages in terms of the compact size, light weight and long durability make wearable electronics an excellent solution in various realizations of platforms for breath analysis and respiration monitoring [3,4,5,6,7,8,9,10,11,12,13,14,15,16,17]. Breath analysis and the monitoring of respiration rate are very important indicators of health state, particularly during the COVID-19 pandemic. Moreover, the monitoring of our breathing can be used as a marker for the detection of various diseases, such as sleep apnea, asthma, and cardiac arrest [3]. For example, an integrated flexible triboelectric-powered wearable sensor for the monitoring of respiratory patterns was reported by Wang et al. [4]. The placement of sensors near the nose was also reported as an approach for breathing rate monitoring by Folke et al. [5] and Al-Khalidi et al. [6]. The respiration rate was also monitored using acoustic sensors mounted on the neck, as reported by Corbishley et al. [7] and Mimoz et al. [8]. However, sensors on the nose and throat are not comfortable for wearing over prolonged periods of time, and especially in public [9]. 

In addition to the above-mentioned realizations, conductive textile technology was also used for the fabrication of sensors for respiration rate monitoring [10,11,12,13,14,15,16,17]. A wearable yarn-based piezo-resistive sensor was reported as a feasible solution for the tracking of the respiration rate by Huang et al. [10]. A realization of a respiration belt based on a textile strain sensor was also reported by Atalay et al. [11]. Inductive fiber-meshed strain and displacement transducers for respiratory monitoring systems were presented by Wijesiriwardana et al. [12]. Textile-based pressure sensors were also investigated as a solution for respiration rate monitoring [13,14,15,16]. Further technology advances, with a trend of reducing price, lead to the embedding of the resistive respiration rate monitoring sensors to a shirt, with readout electronics being placed in a removable box [17].

Despite the fact that numerous research articles have explored various approaches in respiration rate monitoring, to the best of our knowledge, there is still no textile-based respiration rate sensor embroidered onto a facemask. However, the realization of the respiration monitoring systems with an ordinary facemask was explored by Güder et al. [18] and by Zhong et al. [19], as well as special customized facemasks by Tipparaju et al. [20]. A paper-based humidity sensor for respiration monitoring integrated with a facemask was connected with a smartphone through a Bluetooth connection for post-processing and data acquisition. Carbon ink was used for the printing of electrodes [18]. The main limitation of paper-based humidity sensors is the linear response in a very narrow humidity range [21,22]. Moreover, relative humidity-based respiration sensors are still undergoing huge investigation, as flexibility, fast response and recovery speed, and non-toxicity have still not been fully achieved [23,24]. A pressure sensor and readout circuit were integrated with a normal face mask in the study reported by Zhong et al. [19]. However, the main limitations of the presented work are the lack of ability to allow air to pass through the sensor and the complicated fabrication process [19].

With the aim of providing a better overview of the current state of the art and to identify open gaps targeted in our work, the main features and limitations of the reported works are summarized in Table 1.

Advancing the state of the art shown in Table 1, in this paper, we present a portable respiration monitoring system with an embroidered capacitive facemask sensor. To the best of our knowledge, the developed textile-based capacitive sensor, embedded in the ordinary facemask, is the first realization of such a system in the scientific community. The working principle is based on capacitance (permittivity) change due to the humidity change caused by exhaled air. Respiration monitoring by breath air flow analysis offers an advantage when compared to the sensors deployed in the abdomen, chest and throat in terms of the multi-variable monitoring and analysis of temperature, humidity and molecule detection [25]. Our sensor consists of humidity-sensitive hygroscopic polyester thread and silver-coated polyamide conductive thread-based interdigitated electrodes acting as a capacitor. The presented approach is an important advancement in developing eco-friendly and flexible sensors, without expensive raw materials, which is marked in the literature as a task of high importance [26]. Moreover, the capacitive nature of the fabricated sensor is a step forward in lower consumption, when compared to the reported resistive sensors. Initially, the embroidered sensor was characterized in a humidity chamber and with a commercial impedance analyzer. Furthermore, suitable readout electronics, based on a direct interface of the sensor and the microcontroller, were proposed and verified. A smartphone or personal computer (PC) was connected to the readout electronics through a universal serial bus (USB) cable. The same interface was used for power supply, as well as for data acquisition, consequently sending data to the server. Finally, the integrated system was in situ tested with 10 human volunteers. The obtained data showed that the proposed system is capable of accurate and reliable respiration rate monitoring. When compared to the reported works, our realization offers advantages such as the low-cost and portable implementation of the readout electronics (<15 USD), a flexible sensor with a fast response and recovery time, a production cost of less than USD 1, a simple fabrication process, and nontoxicity. Additionally, the power consumption of the developed respiration monitoring system was determined to be as low as 70 mW, confirming the assumption of a low-power operation.

The rest of the paper is organized as follows: the general system architecture and the design and optimization procedures of the capacitive facemask sensor and readout electronics are described in Section 2. Section 3 contains the main experimental results for the characterization of the sensor and readout electronics, as well as their integration in the system for respiration monitoring. Finally, the main achievements and plans for future works are summarized in Section 4.

## 2. Materials and Methods

### 2.1. The System Architecture

**Figure 1:**
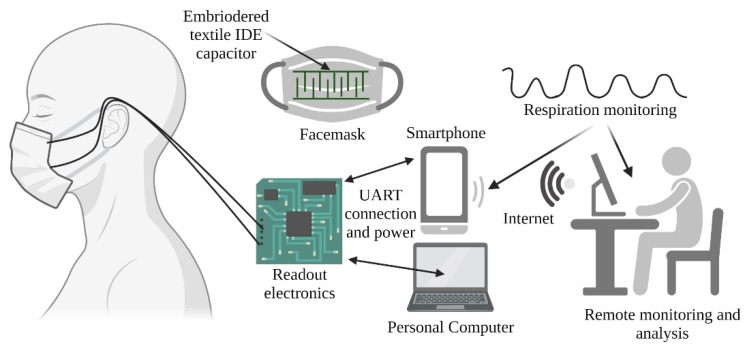
Block schematic of the system for respiration monitoring.

A textile-based capacitor with interdigitated electrodes (IDE) was embroidered onto the inner side of a protective facemask. Capacitor electrodes were connected with copper wires to the readout electronics. To enable the plug and play use of the developed readout electronics with different facemasks, a 3.5 mm connector was used to connect/disconnect wires from readout electronics. Wires were attached to the two capacitor electrodes with rivet buttons. The readout electronic board can be connected through a USB cable to a smartphone or PC, which also acts as the power source for the readout electronics, as well as the unit for data acquisition and display. Furthermore, by using an Internet connection, it was possible to send data to a remote monitoring station. The graphical presentation of the proposed system is given in Figure 1.

### 2.2. The Facemask Sensor

**Figure 2:**
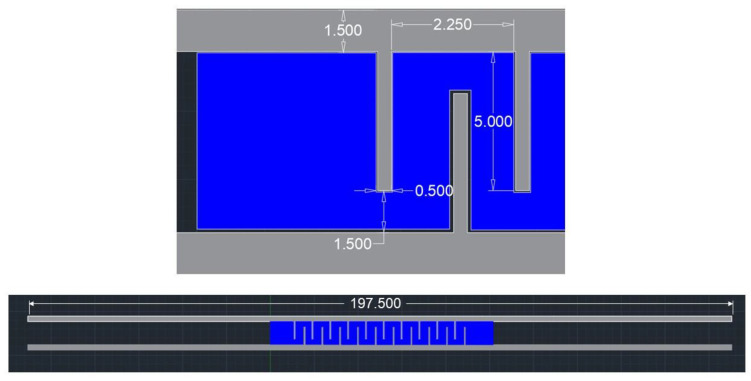
Designed specifications of the embroidered capacitive facemask sensor.

To realize the embroidery of the interdigitated electrode design, the sensor topology was first designed in Autodesk AutoCAD 2021 (Figure 2). As shown in Figure 2, there were 10 fingers per side. The surface area separation from the fingers was 0.2 mm, while the surface area of the hygroscopic layer was 340 mm^2^. Facemask sensor design parameters were selected to be incorporated into a fold of the face mask, before stretching the mask to cover the whole face. The vector graphic file was subsequently imported in a proprietary stitch digitizing software (BasePac 10, ZSK, Krefeld, Germany) in order to be transformed into the machine code bearing stitch information. The hygroscopic thread was blue, while the conductive thread was silver. 

The face masks were created using a technical embroidery machine (JCZA 0109, ZSK Germany) with a minimum axis resolution of 0.1 mm. A water-soluble poly(-vinylalcohol) (PVA)-based backing material (Madeira, North Yorkshire, UK) was applied to the embroidery field. As the design was fabricated on an industrial technical embroidery machine, the embroidery field was very big compared to the size of an individual mask (approximately 75 cm by 90 cm). This means that in order for embroidery to be performed, the mask had to be appropriately attached to the frame, which was the provider of the X and Y axis motion as the needle was static in the position with a hole linking to the bottom thread. Therefore, if any regular fabric was used, a piece of it would be “sandwiched” between the top conductive thread, the mask, and the bottom bobbin thread. This could not only potentially have adverse effects on the performance of the system, but also increase the height of the embroidery, leading to user discomfort. Therefore, a backing material was used instead, which was made from water-soluble PVA. This was an extremely thin material compared to normal fabrics and was used only to provide a secure place for the mask to be attached onto. Then, it could be easily torn off, and left no remains on the embroidered product. The part that was still “sandwiched” between the embroidery layers then gradually dissolved due to the residual humidity of breathing.

The sensing part of the mask was created by using the SilverTech 120 silver-coated polyamide conductive thread produced by Amann Group (Bönnigheim, Germany), with a nominal resistance of less than 530 Ω/m. The conductive thread was made by melt-spinning. This procedure is composed of several steps. Initially, a single stand in the thread is melted, then pressed through spin plates whilst tuning the dosage, successively cooled down, and extended. This procedure leads to the crystalline areas of the polymer sliding off and aligning in the direction of the pulling. In the next phase, the plating of the threads is performed with silver. However, the plating does not yield a uniform silver foil, varying in thickness from 50 nm to 200 nm, as detailed in our previous work [27]. The hygroscopic thread used in between the interdigitated finger structures was the Burmilon 200 weight spun polyester thread produced by Madeira, UK. The underthread (bobbin thread) throughout the design was also supplied by Madeira, UK, and is a generic 150 weight polyester thread.

Due to the fact that embroidery strikes a delicate balance between the top and bottom thread tensions, the conditions and appropriateness of the needle, the tension of the backing fabric on the embroidery field and overall fabric stability, an allowable tolerance of approximately ±0.2 mm was applied to all measurements.

### 2.3. The Readout Electronics

The conventional techniques for an interface with capacitive sensors are based on electrical impedance spectroscopy (EIS) [28]. However, such an approach is not reliable for interfacing with low-value capacitive sensors, as the impedance of the capacitive sensors is very high at low and moderate frequencies. The impedance of the capacitive sensors is reciprocal to the capacitance *C* and angular frequency ω (Z = (*ωC*)^−1^), with a relationship *ω* = 2*πf* between the angular frequency ω and frequency *f*. Therefore, high-frequency measurements of impedance are needed, which increases the required complexity of the measurement device. In addition to this, high-frequency measurements include other parasitic phenomena, such as inductance.

Moreover, for many capacitive sensors, there is no need to perform impedance spectroscopy, as the sensing mechanism is very well understood, and there is a clear relationship between measured parameters and capacitance [29]. For example, the humidity and dielectric constant of the material can be easily monitored with basic capacitance measurement. Additionally, the EIS approach is not easily deployable on low-cost, portable, and microcontroller-based readout platforms. The microcontroller-based readout of capacitive sensors is usually performed via two approaches: (1) capacitance to time conversion and (2) capacitance to voltage conversion. 

Capacitance to time conversion includes the measurement of the time interval of the charging process of the capacitance under test (*C_x_*) connected to the reference resistor (*R*) [30]. Two digital input/output (I/O) pins of the microcontroller are used for the control of the capacitor charging and discharging process, respectively. The time constant of the charging process (*τ = RC_x_*) is the time interval required for the voltage of the capacitor from 0 V to reach 63% of the steady state value (high logic state—*V_OH_*). The implementation approach for capacitance measurement is based on the use of an internal timer for the measurement of the time constant and an analog to digital converter (ADC) for the voltage measurement. 

A voltage equal to 63% of the *V_OH_* is captured with the ADC, and the elapsed time divided by *R* is equal to the value of *C_x_*. However, in the case of capacitors with low capacitance (pF-range, for example), the capacitance to time conversion method needs a high resistance value of the reference resistor. Otherwise, the time constant will not be detectable because typical microcontrollers can measure time very precisely, even in the range of nanoseconds, but usually do not have fast internal ADCs with a sampling rate capable of detecting fast varying voltages and determining the exact value equal to the 63% of the *V_OH_* value. 

Moreover, high-value resistors are known to be subject to thermal noise and tolerance issues. A reduction in the resistance value will reduce thermal noise, but it will also reduce the time constant value. The capacitance to voltage conversion is a possible solution that overcomes the above-mentioned limitations of capacitance to time conversion, as it is based on steady-state voltage measurement.

**Figure 3:**
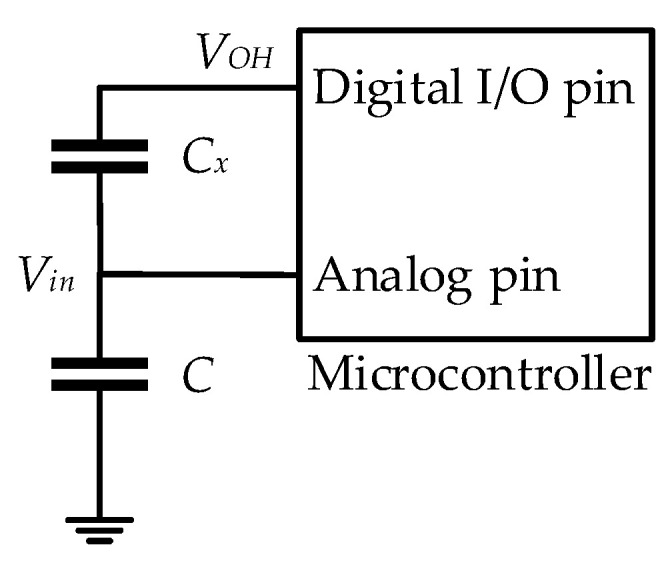
Electrical schematic of microcontroller-based capacitance to voltage conversion.

Capacitance to voltage conversion is based on the capacitive voltage divider circuit [31], formed by an unknown capacitance *C_x_* and a reference capacitor *C*, as shown in Figure 3.

Voltage at the input of the ADC is:

(1) $$V_{in}=V_{OH}\frac{C_{x}}{C_{x}+C}$$

With the obtained value of voltage *V_in_*, measured by the ADC, it is possible to calculate the unknown capacitance as:

(2) $$C_{x}=C\frac{V_{in}}{V_{OH}-V_{in}}$$

The voltage reference of the ADC is usually equal to the value of the *V_OH_*; therefore, it corresponds to the maximum digital word of the ADC, while voltage *V_in_* is:

(3) $$V_{in}\left(n\right)=\frac{n}{N}V_{OH}$$

where *n* is the corresponding digital word of the ADC, and *N* is the number of quantization steps of the ADC. If (3) is combined with (2), the expression of the unknown capacitance calculation becomes:

(4) $$C_{x}=C\frac{n}{N-n}$$

Therefore, the accuracy of the capacitance measurement using capacitance to voltage conversion is determined by the actual value of the reference *C* and ADC accuracy. The typical accuracy of ADCs embedded in microcontrollers is ±2 least significant bits (LSBs), while the actual reference capacitor value is dependent on the used capacitor tolerance, and it can be determined with calibration.

**Figure 4:**
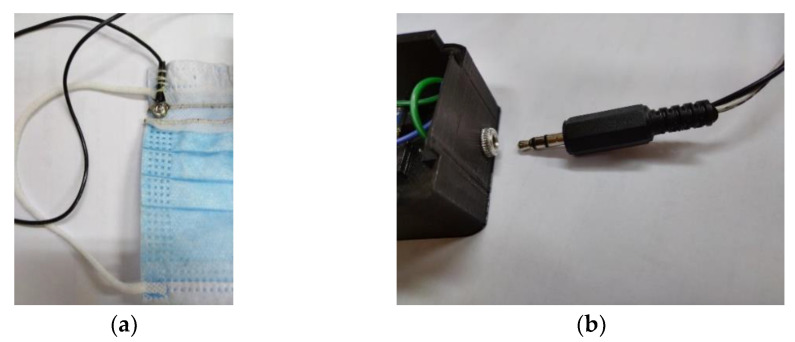
Electrical connections between the readout electronics and the facemask sensor: (**a**) snap buttons on the facemask and (**b**) snap buttons connected to the readout electronics through a copper wires and a 3.5 mm connector.

The connection between the readout electronics and the conductive threads used for the facemask sensor was made using conductive metal snap buttons. Snap buttons were sewn to the facemask (Figure 4a) and soldered to the wires (Figure 4b). The proposed solution showed excellent stability without unwanted detachment. In addition to this, a reliable connection/disconnection could be easily made. 

### 2.4. Data Acquisition System

Data acquisition of the system measurements can be performed by either PC-based software or by smartphone application. In both cases, the application contains a serial communication module and modules for the graphical and textual presentation of the received data. Modules for communications with the remote server can also be included.

### 2.5. Validation of the Obtained Respiration Monitoring Data

Validation of the developed respiration rate sensor is usually performed with one or more of the following four approaches [32]:

1. Artificial validation prototypes;
2. Metronome as a reference;
3. Validation against a reference device;
4. Physical assessment.

Artificial validation prototypes are based on controlled humidity chambers with humidifiers and dry air supply units. The main parameter for such devices is the capability to generate a fast and reliable sequence to replicate human respiration, which is usually not a simple task as the feedback loop requires sensors with a very fast response time. This approach is more common in the case of strain/force sensor-based respiration monitoring systems.

The use of metronome as a reference offers an advantage over the artificial validation method based on the fact that it involves actual human subjects. However, there is no guarantee that the test subjects will exactly follow the requested rate of the metronome, which can introduce an error in evaluation.

Validation against a reference device is also considered as a gold standard for the validation of the new sensors. However, the reference sensor and the sensor under validation must be synchronized and must be worn at the same time. Moreover, the reference sensor also has limited accuracy, which must be taken into account during the validation of the developed sensor. In the case of the commercial respiration rate monitoring devices, some important challenges must be addressed [31]: most commercial systems do not provide information on how the breathing parameter is extracted from the measured values or the duration of the time frame for data collection. The use of the commercial humidity sensors embedded into our facemask was also analyzed. However, the declared response times of available sensors were as low as 4 s (Innovative Sensor Technology, Las Vegas, NV, USA, model HYT 271), 7 s (Amphenol advanced sensors, St. Marys, PA, USA, model CC2D33S-SIP), or higher, which was not fast enough for respiration rate monitoring.

Physical assessment is a method in which the volunteering subject and observing investigator monitor the respiration of the subject. As both count respiration cycles, with cross-validation it is possible to obtain a reference value that presents ground truth, based on which the accuracy of the developed sensor can be determined. We chose this approach in the presented study.

## 3. Experimental Results and Discussion

### 3.1. Sensor Embroidered on Protective Face Mask

**Figure 5:**
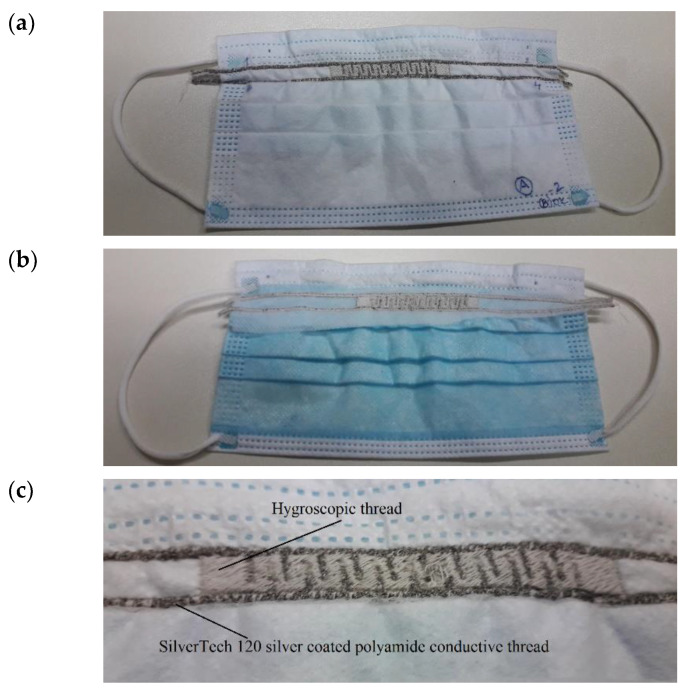
Facemask with embroidered capacitive sensor: (**a**) inner side, (**b**) outer side and (**c**) IDE capacitive sensor.

Water molecules are an essential constituent of respiration, which influence the relative humidity (RH) around our nose and mouth during inhaling and exhaling. Thus, the rapid and accurate determination of water molecules in real time remains a challenge for present-day humidity sensors. The presented face mask sensor (Figure 5), comprising humidity-sensitive hygroscopic polyester thread and silver-coated polyamide conductive thread-based interdigitated electrodes, acts as the capacitor.

By taking into account the cost of the individual mask piece, as well as the other necessary materials, such as the stabilizer fabric and conductive thread, and also considering the necessary analogy for power consumption and equipment maintenance and amortization, the estimation of the production cost of an individual mask is approximately USD 1. However, this cost does not include any marketing actions and is heavily dependent on fluctuations in the global noble metal and energy markets.

Challenges in the practical use of the developed sensor were primarily expected to be related to the triboelectric effect and movement artefacts. 

The triboelectric effect was considered by the careful design of electrodes with a very limited area in contact with skin. Moreover, the contact area between the skin and sensor does not change over time, which is in accordance with the assumption that the transient process will occur at the very beginning, causing charge transfer, without a significant impact in the later phase of the measurement.

Our current realization was directed towards home-medical testing applications, rather than realization with mobility, flexibility and liberty of movement. We do not see this as a highly limiting factor, as many commercial devices require operation under strict conditions. However, as our sensor is capacitive in nature and it does not rely on movements of body organs, we also expect high potential in moving applications. 

With the aim of analyzing the increase in the weight of the facemask when the capacitive sensor is embroidered into it, we performed measurements with a digital scale for accuracy (Colossus CSS-3500, Dandong, Liaoning, China). The obtained results are 2.87 g without the sensor and 3.97 g with the sensor. Therefore, the increase in weight is approximately 1.1 g, confirming the assumption of the lightweight property of the developed sensor. 

The embroidered structure shown in Figure 5 was evaluated for humidity testing under normal laboratory conditions. The instrumentation used for this testing involved the Owlstone V-OVG system (Owlstone Ltd., Cambridge, UK) equipped with a closed humidity chamber and a chemical impedance analyzer (IM3590, Hioki, Japan). Humidity-sensitive threads were found to be susceptible to moisture, which affected the capacitance values, producing different response signals within a wide relative humidity (RH) range from 36% to 78%. The principle of the fabricated capacitive-type face mask sensor regarding the humidity measurement was established based on the dielectric property changes in the sensing thread among electrodes. Upon the interaction of water molecules, the permittivity of the sensing threads increased due to the relatively high permittivity of water, which eventually caused the capacitance values to increase [33]. Thus, relative humidity was evaluated by analyzing the increase in the capacitance values of the facemask sensor. The hygroscopic thread used in this study was polyester-based. The reason for the behavior of capacitance change can be further ascribed to the presence of the polar carbonyl group (*C*=O with stretching vibrations at around 1700 cm^−1^) in the polyester thread, which helped in the easy absorption of moisture [34]. Thus, relative humidity was evaluated by analyzing the increase in the capacitance values of the facemask sensor.

**Figure 6:**
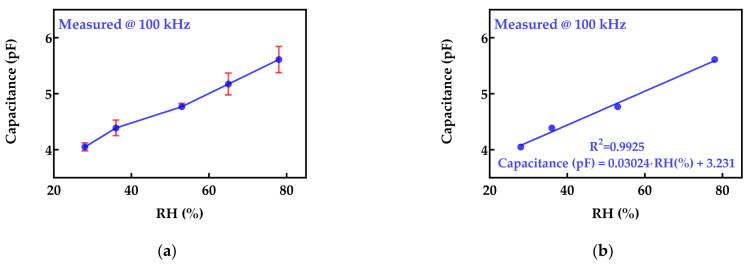
(**a**) Increase in capacitance of the face mask sensor with the increase in relative humidity at 100 kHz (mean values and error bars for three times repeated measurement); (**b**) results of linear regression analysis.

The capacitive response of our facemask sensor with the increase in relative humidity at 100 kHz is shown in Figure 6a. Measurements were repeated three times; therefore, the mean values and standard deviation were calculated. It can be seen that the variation of the capacitance was obtained in the range from 4.05 pF to 5.6 pF. Therefore, the sensor capacitance increased by 28% when the relative humidity changed from 28% to 78%. Using linear regression analysis for four RH values (28%, 36%, 53%, and 78%) in the given range, it was possible to obtain the calibration curve:

(5) Capacitance (pF)=0.03024·RH(%)+3.231

Using the measured value that was included in the calibration process (65% RH) and Equation (5), the expected sensor capacitance was calculated as 5.19 pF, while the measured value was 5.17 pF. Therefore, a relative error lower than 0.5% was obtained. Moreover, the coefficient of determination (*R*^2^) was calculated as 0.9925. The plot of the measured and fitted values is shown in Figure 6b.

Based on the obtained results, the proposed facemask prototype was evaluated as reliable and was subsequently applied for real-time respiration monitoring. With the optimized readout electronics of the sensor, the overall system was promising for utilization in personal healthcare monitoring applications.

In general, single-use face masks are created from two or three layers of plastic fabric (usually polypropylene) and nylon ear hooks. The hydroscopic layer we added was also made from plastic (polyester)-based thread. Such plastics tend to be relatively inert and exhibit excellent resistance to degradation due to external ambient conditions, except for exposure to UV light. However, this exposure causes adverse effects only after a prolonged period of time, which greatly surpasses the maximum usable time of our embroidered facemasks. Now, with respect to the conductive thread, which is a commercially available silver-coated polyamide thread, once again, its plastic core is expected to have excellent stability towards environmental condition changes, while its silver coating may degrade only with respect to oxidation on its outer part, but once again, at a time that greatly surpasses the usable time of the product, and with a minimal effect on the conductivity of the thread. This behavior was experimentally confirmed with very similar results for the same facemask in a number of tests over one month.

### 3.2. The Readout Electronics

**Figure 7:**
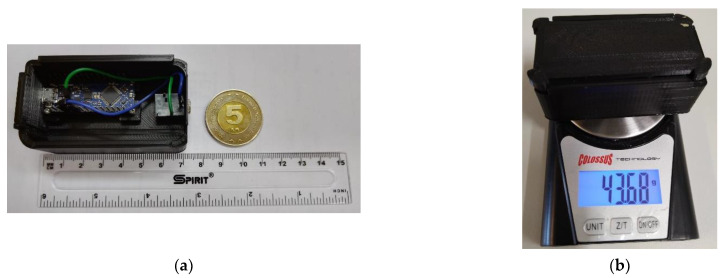
(**a**) The hardware layout of the readout electronics; (**b**) the weight of the final unit.

We used a microcontroller board with the ATmega328P microcontroller. The internal ADC has a 10-bit resolution (1024 quantization steps). With a 16 MHz clock speed, the prescaler of ADC clock was set to 1/128. Therefore, as each conversion takes 13 ADC clocks, the maximum sampling frequency can be 9615 Hz [35]. The used microcontroller board is a very economical solution, costing less than USD 15 [36]. The microcontroller board is placed in the custom-made 3D-printed protective case. The hardware layout of the readout system is shown in Figure 7a. A ruler next to the device and a five euro coin are also shown in Figure 7a. The developed device is intended to be worn throughout the entire duration of the testing (which takes approximately 2 min). As a result, the lightweight property was very important to achieve. The total weight of the final unit, composed of the microcontroller and the 3D-printed case, is less than 44 g (Figure 7b).

**Figure 8:**
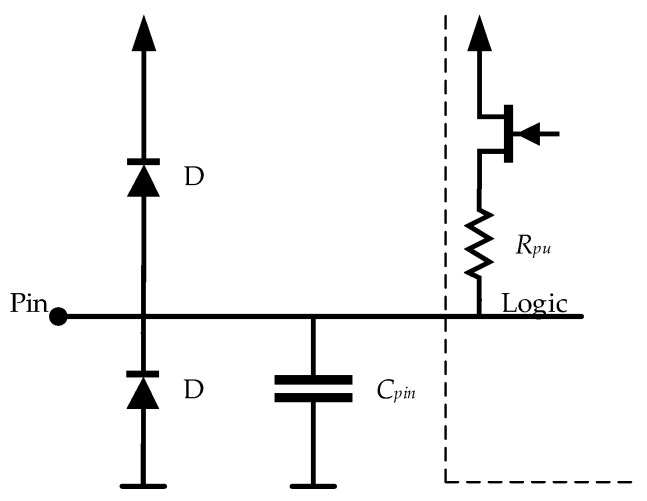
I/O pin equivalent schematic of the ATmega328P [35].

As the ATmega328P microcontroller has internal capacitance (*C_pin_*) associated with each input/output (I/O) pin, as shown in Figure 8, our approach is to use that capacitance as the reference capacitor, instead of adding an external one. Such an approach reduces parasitic phenomena, as well as the overall price and dimensions of the readout system.

However, the exact value of the *C_pin_* capacitance is not given in the datasheet, as we measured the capacitance of two discrete ceramic capacitors (27 pF ± 10% and 47 pF ± 10%) using a commercial impedance analyzer, IM7585 (Hioki, Japan), and the IM9202 test fixture (Hioki, Japan). The test frequency was set to 10 MHz. The obtained impedance modulus (Z), phase angle (θ) and series capacitance (*C_s_*) are shown in Table 2.

**Figure 9:**
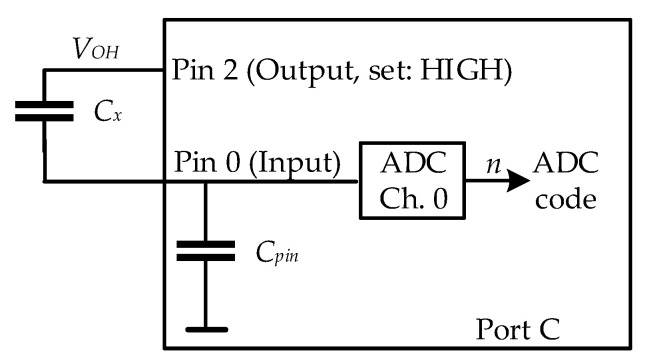
Direct interface of the measured capacitance with the microcontroller.

After that, the aim was to determine the input capacitance on pin 0 of port C, as it is also channel 0 of the internal ADC. Our approach is to use that capacitance as the reference capacitor, as shown in Figure 9. The same capacitors from a previous experiment were connected between port C pins 0 and 2 of the ATmega328P microcontroller, and the ADC value at pin 0 of port C was obtained. Pin 2 of port C was defined as the output and set to high. 

The measurements were repeated 100 times. Obtained average values are close to 672 and 544, as shown in Table 3. 

Low standard deviations (SD) indicate that measurements are stable. By substituting values of 672 for 29.53 pF and 544 for 49.12 pF in Equation (4), it was possible to calculate *C_pin_*. With the obtained values, as shown in Table 3, the average value for *C_pin_* was determined as 25.83 pF ((25.99 + 25.66)/2). Therefore, the capacitance of our reference capacitor was further used as 25.83 pF.

Using Equation (4), it is also possible to calculate the nominal measurement range (*C_min_, C_max_*) with the given configuration:

(6) $$C_{max}\left(n=N-1=1023\right)=C_{pin}\frac{n}{N-n}=1023\times C_{pin}$$

(7) $$C_{min}\left(n=0\right)=C_{pin}\frac{n}{N-n}=\frac{0}{1023}\times C_{pin}=0$$

However, owing to the impact of the power supply fluctuations and ±2 LSB uncertainty of the ADC, a range from 10% to 90% of the full scale was considered. Therefore, an effective measurement range could be from 2.79 pF to 348.19 pF.

Our primary objective was the development of the respiration monitoring system with a low-cost but reliable sensor, which is disposable and eco-friendly, while the readout electronics remained a separate device that could be shared between various users. Despite the fact that our system is composed of some bulky elements, such as a laptop or mobile phone, those elements are multi-use, have a one-time cost and are very commonly used. When compared to the wearable systems with flexible print out readout electronics, our approach offers a lower cost, multiple uses, and the safe disposal of used sensors.

### 3.3. Implementation of Data Acquisition System

**Figure 10:**
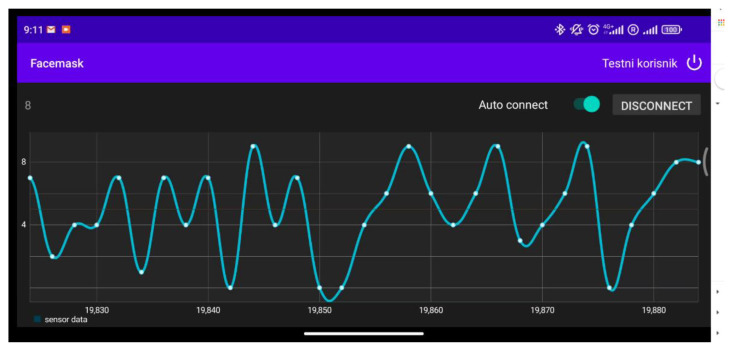
The screenshot of the developed facemask application.

A screenshot of the main screen of the developed facemask application for the Android™ operating system is shown in Figure 10. After starting, it shows a login screen requiring a username and password, which is a mechanism for enhanced security in cases of remote data collection, as it prevents unauthorized access to the data collection process. The main screen is composed of a button for establishing a connection with the current status label (connect/disconnect), a slider for the auto connect option in which the application scans the USB port to check if connected peripheral has an appropriate FTDI chip, and a main part for the graphical presentation of the received data. In the background, the application sends data to the remote server for storage and further processing.

### 3.4. The Respiration Rate Monitoring

Initially, we performed the measurement of the facemask sensor capacitance over a 60 s period, with the aim of determining the stability over a time interval that would be further used. A facemask with an embroidered sensor was connected to the readout electronics and placed on the office table. As the duration of this condition was relatively short, and there were no humidity sources near to the mask, it was expected that minimal capacitance changes would occur.

Capacitance values over time, *C*(*t*), were stored, and finally, the relative change in capacitance over time compared to the initial value *C*_0_ was calculated:

(8) $$\Delta C\left(t\right)=\frac{C\left(t\right)}{C_{0}}$$

**Figure 11:**
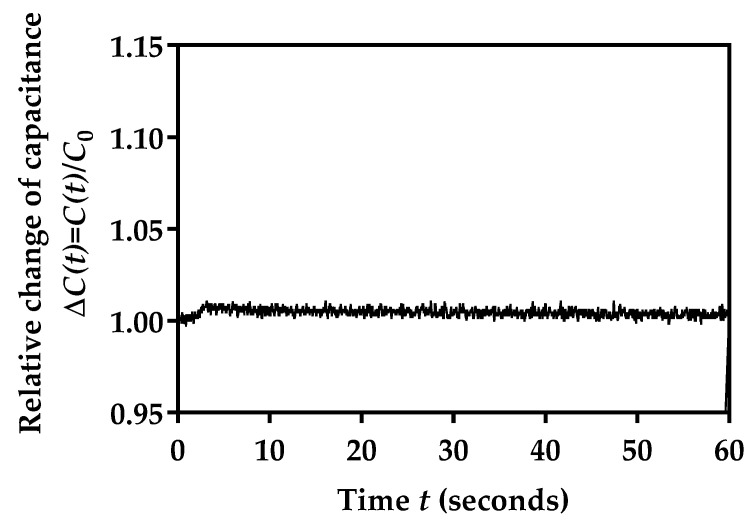
Relative capacitance changes in the fabricated facemask sensor left on the office table without humidity sources.

The value for *C*_0_ was determined as the first value obtained at the beginning of the measurement. As the value for *C*_0_ can slightly differ from one subject to another (given how the facemask is being worn due to the face constitution, which causes different ratios of exposure to environmental and exhaled air, as well as due to different levels of bending), this parameter was only used for the calculation of the relative change in sensor capacitance over time, rather than using its absolute value. Such an approach reduces the possibility for wrong data interpretation. The measured values are shown in Figure 11. As shown in Figure 11, fluctuations were lower than 1%, indicating the very good stability of the sensor readout values.

**Figure 12:**
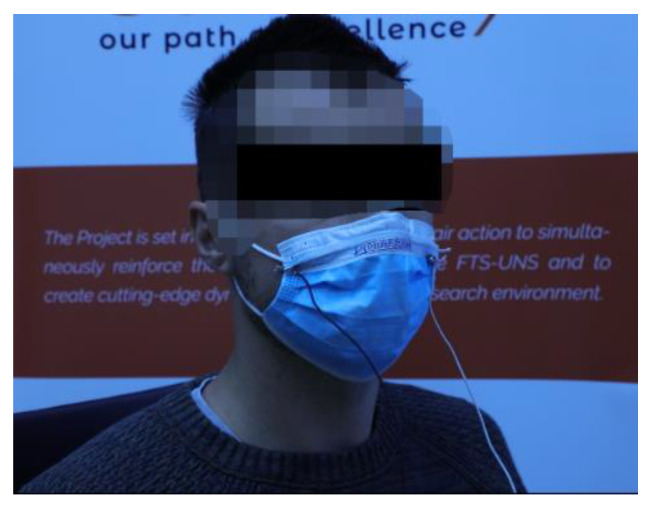
A volunteer wearing facemask with sensor.

For testing purposes, a healthy volunteer subject was also included. The volunteer signed informed consent before the tests. The subject was instructed to sit in a chair and rest for approximately 3 min, which was followed by tests. A volunteer wearing a facemask with the embroidered capacitive sensor is shown in Figure 12.

The protective facemask equipped with the embroidered sensor was connected to the described readout electronics, which were subsequently connected to a laptop via a serial communication terminal. 

To provide a better overview of the sensing performances and to make a clear distinction between respiration phases, the initial testing procedure was defined as follows: a volunteer was in the sitting position and was instructed to hold his breath for 5 s, then to take five breaths, after that to hold his breath for 20 s, and finally to take another five breaths. The end of this protocol was followed by taking the facemask off the volunteer and stopping the recording.

**Figure 13:**
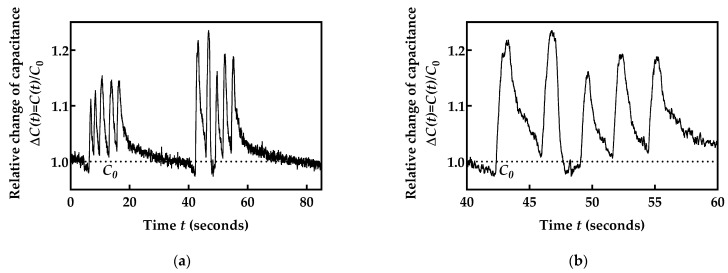
(**a**) Relative capacitance changes in the fabricated facemask sensor for breathing monitoring; (**b**) enlarged portion of a shorter time period.

Capacitance values over time, *C*(*t*), were stored, and the relative change in the capacitance over time compared to the initial value *C*_0_ was calculated using Equation (6). The value for *C*_0_ was 5.24 pF, which was determined as the average value of samples obtained during the time interval of 3 s of the initial breath holding. The obtained capacitance values are in compliance with values measured using a commercial impedance analyzer (Figure 6). As shown in Figure 13, a clear distinction between breath holding and regular breathing phases can be observed. It can also be seen from Figure 13 that, after sensor exposure to the humidity increase during the exhalation phase, the sensor capacitance also increased. In the next phase, the breath holding phase was active and it was observed that the sensor capacitance also decreased and returned to the initial value as it was before the humidity increase (1.0). The same response was achieved in repeated exhalation/inhalation phases.

**Figure 14:**
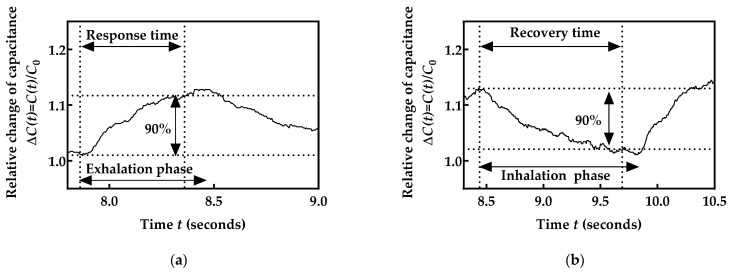
Determination of the response time during (**a**) exhalation phase and (**b**) inhalation phase.

With the aim of verifying whether the fabricated sensor can meet the respiratory rate monitoring requirements, the response and recovery time were determined. The response time was determined as the time interval required to reach 90% of the final value during the exhalation phase. The recovery time was determined as the time interval required so that the sensor value decreases by 90% of the initial value during the inhalation phase. As shown in Figure 14a, the duration of the analyzed exhalation phase was 0.55 s (from 7.86 s to 8.44 s), while the sensor reached 90% of the final value during the exhalation phase for 0.5 s (8.36 s), which is 91% of the exhalation phase duration. Similarly, the duration of the analyzed inhalation phase was 1.39 s (from 8.44 s to 9.83 s) (Figure 14b), while sensor capacitance decreased by 90% of the initial value for 1.26 s (at 9.70 s), which is also close to the 91% of the inhalation phase duration. These results show the feasibility of the developed system for respiration monitoring. The sensor response and recovery times are low enough to track the dynamics of respiratory rates. Furthermore, as the normal respiration rate is composed of inhalation for 1 to 1.5 s and exhalation for 1.5 to 2 s [37], it is clear that the developed system meets the requirements for real-time and continuous respiration monitoring. 

It is also important to emphasize that the developed sensor followed a certain breathing rate in the test presented in Figure 13 and Figure 14; however, the presented response and recovery time were not determined for the step function humidity input and, therefore, they should not be taken as absolute values. With the aim of conducting a more comprehensive analysis of the sensor response, in Section 3.5, we observed the response to different breathing rates.

### 3.5. Performance Testing for Three Different Breathing Rates

**Figure 15:**
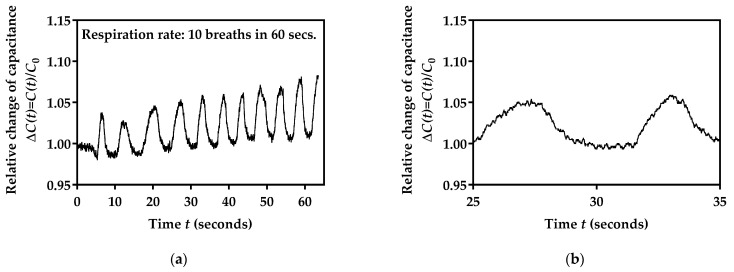

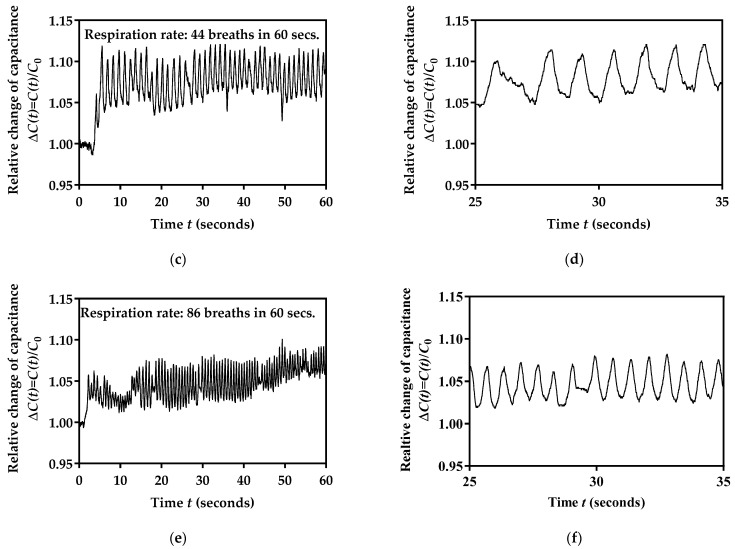
Measured sensor’s capacitance over a 60 s test in the case of (**a**) 10 breaths, (**c**) 44 breaths, (**e**) 86 breaths, and enlarged details of a 10 s period in the case of (**b**) 10 breaths, (**d**) 44 breaths, (**f**) 86 breaths.

In order to obtain a better performance overview of the developed respiration monitoring system, another set of tests with different breathing rates was performed. The volunteer followed the same procedure as described in Section 3.4, but he was instructed to perform three different respiration rates. The exact number of breaths was confirmed by physical assessment (counting by the subject, as well as a supervising researcher). First, 10 breaths were counted for 50 s, followed by two increased respiration rates (44 and 86 breaths), as shown in Figure 15. For clarity, on the right side of the column, enlarged details of three breathing rates are shown for 10 s.

Figure 15 shows that the designed facemask sensor can provide information about the nature of the breaths being taken. For example, at higher breathing frequencies (Figure 15e,f), the signal seemed to be smaller for each breath. This can be explained as the relative change in the capacitance is smaller for faster exhalations, because shallower breaths may draw up less moisture from the lungs, leading to smaller changes in humidity and, consequently, capacitance changes. The importance of this is the possibility to differentiate between shallow and deep breathing, as well as the rate of breathing, which could be very important for various medical diagnostic applications, such as asthma and emphysema, or monitoring lung capacity as a first preliminary indication of COVID-19 infection. The presented system enables the measurement to be performed at home, and the subject can send the recorded signal to the medical expert for detailed analysis and adequate therapy assessment.

### 3.6. Performance Testing for 10 Volunteers

**Figure 16:**
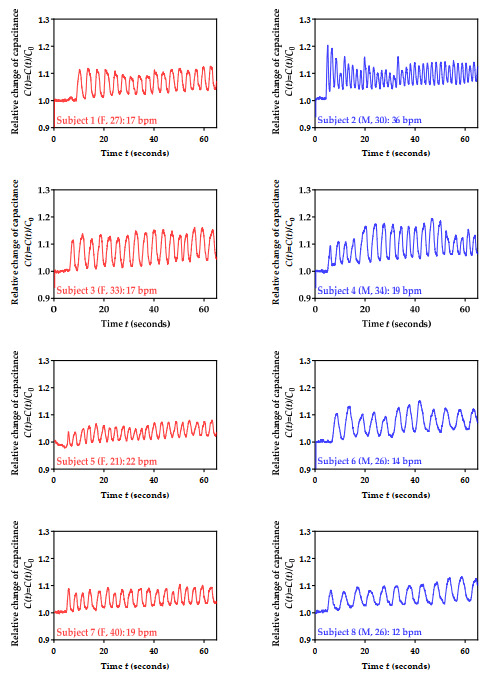

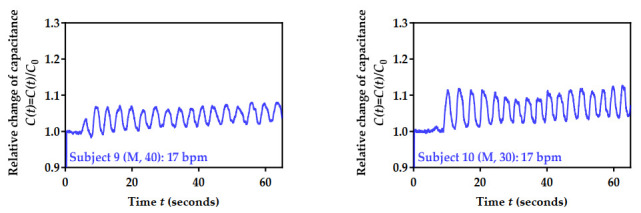
Measured relative change in the sensor’s capacitance for 10 subjects.

In the next step of the validation process of the developed sensor, we performed the respiration monitoring of 10 subjects. Of the 10 volunteers, 6 were males (M) aged 31 ± 5 years, while 4 were females (F) aged 30 ± 8 years. The subjects were instructed to sit in a chair and rest for approximately 3 min, which was followed by testing. The test was composed of initial breath holding for approximately 5 s, followed by 60 s of normal breathing. The obtained results (normalized capacitance values over time) and estimated number of breaths per minute (bpm) are shown in red for females and blue for males in Figure 16. The ages of the subjects are indicated in brackets, in addition to the F/M. The obtained bpm values were confirmed through physical assessment. 

As shown in Figure 16, the data showed very good reproducibility between tests. In addition to that, some interesting data patterns could be recognized. For example, female subjects had a smaller overall increase in capacitance during respiration, which can be attributed to their physiology and smaller lung capacity. The female group also had a very similar breathing rate, with a much smaller standard deviation in bpm value (2) when compared to the male group (8). Within the latter, it can be seen that older males had a smaller overall increase in capacitance during respiration. However, it is difficult to claim that obtained results can be generalized, as a much larger number of volunteers is required for the adequate analysis and proper training of a machine learning model. Moreover, the obtained results can only be linked to the physiological conditions and breathing rates of volunteers at the moment of testing.

Sensor characterization was also performed in terms of *C*_0_ determination for all 10 volunteers. The obtained mean value was 5.19 pF, with a standard deviation of 0.31 pF.

### 3.7. Power Consumption

**Figure 17:**
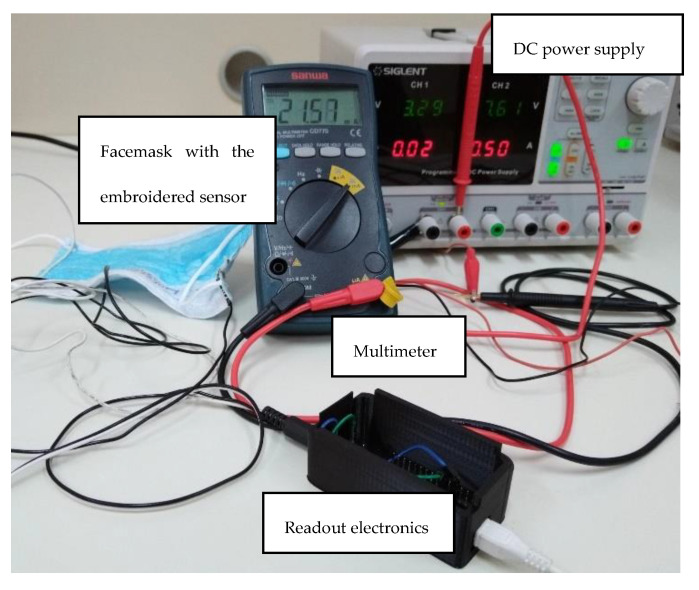
Experimental setup for power consumption determination.

We also observed the energy consumption of the developed system (Figure 17). The microcontroller board with the connected sensor was powered through the USB port using a Siglent SPD3303C generator. The current consumption was monitored using a Sanwa CD770 multimeter. The current consumption of the microcontroller board during operation at 5 volts was 40 mA in total, which corresponds to the power consumption of 200 mW. However, in the case of the 3-volt operation, the current consumption was 22 mA, which corresponds to a very low power consumption of 73 mW, enabling use over a prolonged period of time. For example, if we assume the power supply with the Panasonic NCR18650PF battery (capacity 2750mAh) and a required load current of 22 mA, using the formula:

(9) $$Estimated\;run\;time=\frac{Capacity\;\left(\mathrm{mAh}\right)}{Load\;current\;\left(\mathrm{mA}\right)}=\frac{2750}{22}=125\;h$$

the run time is estimated to be more than 5 days. However, in practical applications, the exact run time must be determined by an actual test, rather than a calculation, as Equation (9) does not include the battery state of health analysis, and assumes 100% efficiency without internal battery losses. 

### 3.8. Mechanical Stability of Sensor Capacitance during Bending

**Figure 18:**
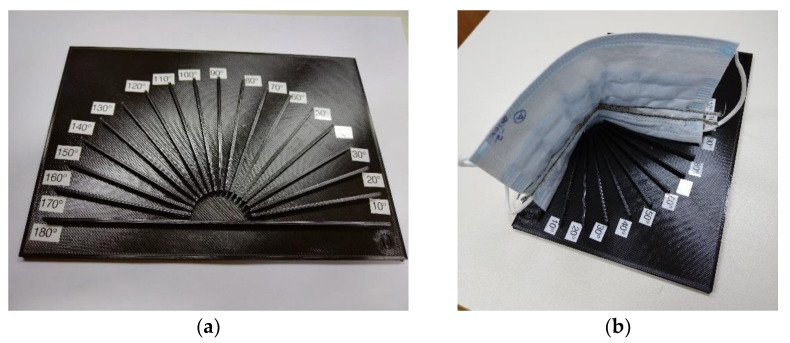
(**a**) Three-dimensionally printed component for facemask bending tests; (**b**) facemask placed in holder with a 90-degree bending angle.

The bending of the facemask during wear may have an impact on sensor capacitance. A small decrease in sensor capacitance may be expected due to the decrease in the effective area between the interdigitated fingers during bending. With the aim of obtaining sensor capacitance at various bending angles, we made a custom, 3D-printed holder, as shown in Figure 18a. This enabled the reliable and repeatable testing of the embroidered facemask sensor, as shown in Figure 18b.

**Figure 19:**
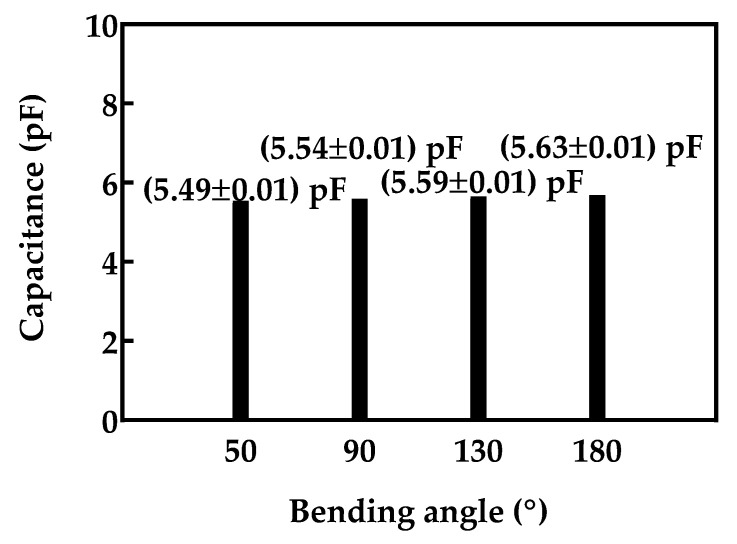
Sensor capacitance for different bending angles.

Measurements were performed for four different angles: 180° (no bending; facemask is fully stretched out), 130°, 90° and 50°, for a period of 30 s. The mean values and standard deviation were calculated. The obtained results, as shown in Figure 19, confirm a very small decrease in sensor capacitance (approximately 1% decrease per 40° bending angle), and very low standard deviation (less than 0.18%).

Sensor capacitance was also monitored over 10 bending cycles from the normal position without bending (180°) to the bending angle of 50°. Capacitance values over time *t* during 10 bending cycles, *C*(*t*), were stored and normalized to the initial value without bending (value at 180°):

(10) $$\Delta C_{m}\left(t\right)=\frac{C\left(t,\;Bending\;angle\right)}{C_{0}\left(Bending\;angle=180{}^{\circ}\right)}$$

**Figure 20:**
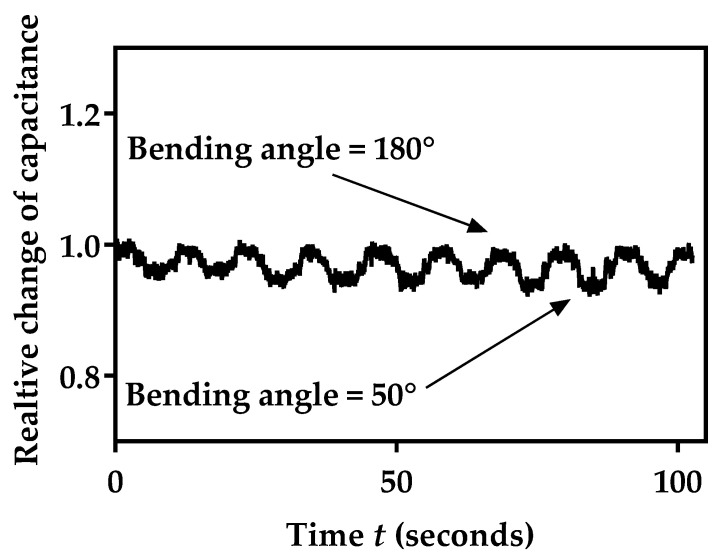
Relative change in sensor’s capacitance during 10 bending cycles.

The obtained values, as shown in Figure 20, indicate very small fluctuations during bending, as well as stable baseline capacitance *C*_0_.

### 3.9. Performance Comparison of the Proposed Device with the State of the Art

The results of the comprehensive performance analysis of the developed system are presented in the previous subsections. With the aim of providing a better overview of the significance of our work and the main contributions to the field, we performed a performance comparison of our solution with the relevant recent works.

When compared to the respiratory belts, which are come in the form of piezo-resistive sensors [10,17], resistive strain sensors [11], inductive strain sensors [12], textile capacitive force sensors [13], or bio-triboelectric nanogenerators [38,39], the advantages of our system are primarily as follows: simpler readout electronics and elimination of abdominal and chest breathing interference because our system is based on breath air analysis. The same limitation of the textile-wearable capacitive sensor is also resolved [16]. 

Moreover, our system successfully recorded respiration rates from 10 bpm to 88 bpm, which was also in the range of interest of reported works: 10 and 30 bmp [10]; 8, 12 and 24 bpm [11]; 10 bpm [12]; 13–25 bpm [40]; 20–24 bpm [41]; 39 bpm [42]; and 60 bpm [43]. Therefore, our system reached a much higher upper limit, indicating that it can be potentially deployed in user-alerting applications.

Accurate respiration rate monitoring requires a high sampling rate and reliable data acquisition. Our system has a sampling rate of 250 Hz, which is much higher than the state of the art: 2 Hz [16], 6 Hz [44], 10 Hz [11,12], and 100 Hz [13], and in the range of the commercial OC-driven NI PCI-6024 A/D card, which has the same sampling rate [10].

Paper-based humidity sensors have a linear response in a very narrow humidity range [18]; however, our sensor has a coefficient of determination equal to 0.8514 for a relative humidity range from 36% to 78%.

When compared to the flexible pressure sensor embroidered into the facemask [19], our solution eliminates issues such as the lack of ability to allow air to pass through the sensor and the complicated fabrication process.

Finally, our sensor has a significantly lower weight (1.1 g) when compared to the solution based on the differential MEMS pressure sensor and Venturi tube (160 g) [20].

## 4. Conclusions

Remote respiration monitoring is a very important aspect of various biomedical applications, as well as in the everyday lives of many people. With the rapid development of emerging technologies such as the e-textile paradigm, the development of personalized healthcare systems became possible with user-friendly components. Despite the fact that numerous research articles have explored various approaches to respiration rate monitoring, there is still a growing need for the development of lightweight, disposable, eco-friendly and flexible sensors, without expensive raw materials, and with low power consumption. 

Our work in the field of the development of textile-based capacitive sensors with appropriate readout electronics provided the ordinary facemask with new features. We successfully demonstrated that it is possible for the ordinary facemask to be a part of reliable respiration monitoring in real time. Our key achievements are as follows: (1) the first reported the successful integration of the embroidered textile capacitive sensor with the facemask; (2) the lightweight property of the developed sensor with an increase of only 1.1 g in the facemask weight with the integrated sensor; (3) fast response and recovery times; (4) portable and reliable readout electronics that allow respiration data exchange between the sensor and mobile phone or PC; and (5) the developed software application for mobile phones with an Android operating system. As we successfully demonstrated the functionality of our prototype in an operational environment, the estimated TRL of our developed system is TRL 6.

The developed solution has a huge potential impact, especially in the ongoing global COVID-19 pandemic, because it enables respiration monitoring at home and recorded signals to be sent to clinicians.

Our future work will be directed towards the realization of readout electronics on a miniaturized footprint in a form compatible for attachment to facemasks and wireless data transmission (using Bluetooth Low Energy (BLE) and near-field communication (NFC)) to a mobile phone, as well as investigating another biomedical application, such as bacteria presence detection (for example, *Helicobacter pylori*) from exhalation, using a similar system to that demonstrated here. Our current realization was directed towards home-medical testing applications (point-of-care testing concept), rather than realization with mobility, flexibility and liberty of movement. We will explore the performance of our sensor in applications including movement in our further studies, including commercial sensors for environmental monitoring and an accelerometer for noise reduction generated during walking.

## Figures and Tables

**Table 1:** State of the art in field of respiration monitoring sensors.

Reference, Year	Type of the Sensor	Mounting Details	Reported Limitations
[10], 2008	Piezo-resistive sensor	Respiratory belt	Relatively complex readout circuit; large size; change in conditions between skin and electrodes over time causes changes in sensor response; the sensor positioning is very restricted due to sensitivity to the chest and abdominal breathing; limited sampling rate.
[11], 2015	Weft-knitted resistive strain sensor		
[12], 2006	An inductive fiber meshed strain sensor		
[13], 2014	Textile capacitive force sensor		
[17], 2013	Piezoresistive sensor		
[16], 2013	Textile wearable capacitive sensor	Two electrodes are fixed on the inner anterior and posterior sides of a T-shirt.	The sensor positioning is very restricted due to sensitivity to chest and abdominal breathing; limited sampling rate.
[18], 2016	Paper-based moisture sensor	Sensor embedded in the facemask.	Linear response in a very narrow humidity range, fast response and recovery speed, and non-toxicity are still not fully reached.
[19], 2021	Flexible pressure sensor	Sensor embedded in the facemask.	Lack of ability to allow air to pass through the sensor and complicated fabrication process.
[20], 2020	Differential MEMS pressure sensor and Venturi tube	Sensor embedded in the facemask with a robust plastic case.	Complex mechanical structure; total weight of the mask device is 160 g; solution based on the commercial sensors has limited level of innovation.

**Table 2:** Measured values of ceramic capacitors.

Nominal Value	Z (Ω)	θ (deg)	*C_s_* (pF)
27 pF ± 10%	539.027	−89.931	29.5264
47 pF ± 10%	324.034	−89.958	49.1168

**Table 3:** Measured values of ceramic capacitors.

Nominal Capacitance	Measured with HIOKI	Average ADC Value	SD of ADC Value	*C_s_* (pF)
27 pF ± 10%	29.53 pF	671.94	0.24	25.99
47 pF ± 10%	49.12 pF	543.86	0.35	25.66

## Data Availability

Data are available upon reasonable request.
